# Enhanced Prostacyclin Synthesis by Adenoviral Gene Transfer Reduced Glial Activation and Ameliorated Dopaminergic Dysfunction in Hemiparkinsonian Rats

**DOI:** 10.1155/2013/649809

**Published:** 2013-04-03

**Authors:** May-Jywan Tsai, Ching-Feng Weng, Nien-Chu Yu, Dann-Ying Liou, Fu-San Kuo, Ming-Chao Huang, Wen-Cheng Huang, Kabik Tam, Song-Kun Shyue, Henrich Cheng

**Affiliations:** ^1^Neural Regeneration Laboratory, Department of Neurosurgery, Neurological Institute, Taipei Veterans General Hospital, Taipei 11221, Taiwan; ^2^Center for Neural Regeneration, Neurological Institute, Taipei Veterans General Hospital, Taipei 11221, Taiwan; ^3^Institute of Biotechnology, National Dong Hwa University, Hualien 97401, Taiwan; ^4^School of Medicine, National Yang-Ming University, Taipei 11221, Taiwan; ^5^Institute of Biomedical Sciences, Academia Sinica, Taipei 11529, Taiwan; ^6^Department of Pharmacology, National Yang-Ming University, Taipei 11221, Taiwan

## Abstract

Prostacyclin (PGI_2_), a potent vasodilator and platelet antiaggregatory eicosanoid, is cytoprotective in cerebral circulation. It is synthesized from arachidonic acid (AA) by the sequential action of cyclooxygenase- (COX-) 1 or 2 and prostacyclin synthase (PGIS). Because prostacyclin is unstable *in vivo*, PGI_2_ analogs have been developed and demonstrated to protect against brain ischemia. This work attempts to selectively augment PGI_2_ synthesis in mixed glial culture or in a model of Parkinson's disease (PD) by direct adenoviral gene transfer of prostacyclin biosynthetic enzymes and examines whether it confers protection in cultures or *in vivo*. Confluent mixed glial cultures actively metabolized exogenous AA into PGE_2_ and PGD_2_. These PGs were largely NS398 sensitive and considered as COX-2 products. Gene transfer of AdPGIS to the cultures effectively shunted the AA catabolism to prostacyclin synthesis and concurrently reduced cell proliferation. Furthermore, PGIS overexpression significantly reduced LPS stimulation in cultures. *In vivo*, adenoviral gene transfer of bicistronic COX-1/PGIS to substantia nigra protected 6-OHDA- induced dopamine depletion and ameliorated behavioral deficits. Taken together, this study shows that enhanced prostacyclin synthesis reduced glial activation and ameliorated motor dysfunction in hemiparkinsonian rats. Prostacyclin may have a neuroprotective role in modulating the inflammatory response in degenerating nigra-striatal pathway.

## 1. Introduction

Parkinson's disease (PD) is characterized by the progressive degeneration of nigrostriatal dopaminergic (DA) accompanied with inflammatory changes leading to activation of microglia and astrocytes [1]. The substantia nigra (SN) of the brain is particularly rich in microglia [2, 3]. In addition, dopaminergic neurons in the SN have a reduced antioxidant capacity, rendering them vulnerable to a variety of insult. Inflammatory responses are also associated with the effects of dopaminergic neurotoxins, 6-hydroxydopamine (6-OHDA) or 1-methyl-4-pheny-1,2,3,6-tetrahydropyridine (MPTP). Activated microglia and DA cell loss were found in the primate SN years after MPTP treatment [4]. In the striatum and SN of 6-OHDA-lesioned rat brains, prominent microglial activation was detectable weeks after the lesion [5]. Intranigral injection of LPS in rats also resulted in microglial activation and degeneration of the DA system [6, 7]. Whether microglial activation protects or exacerbates neuronal loss is presently debated, though most studies suggest that activated microglia exerts a toxic effect on neurons. 

The prostanoids, a naturally occurring subclass of eicosanoids, are lipid mediators generated through the oxidative metabolism of 20-carbon fatty acids (eicosa is Greek for 20), primarily arachidonic acid (AA). Prostaglandins (PGs), synthesized from AA by cyclooxygenases (COXs), have diverse biological actions by working as local mediators. In the central nervous system (CNS), PGs maintain important functions as retrograde synaptic messengers and as early mediators of neuronal injury. The levels of PG production are mediated by the expression and activity of COX. COX exists in two distinct isoforms, constitutive COX-1 and inducible COX-2. COX-2 is responsible for the increased production of prostanoids during inflammation and stress [8, 9]. In the brain, COX-2 is constitutively expressed and is also the dominating COX isoform [10, 11] that mainly produces PGE_2_ and PGD_2_ [12, 13]. Prostacyclin (PGI_2_), synthesized by sequential action of COX and prostacyclin synthase (PGIS), is a potent endogenous inhibitor of platelet aggregation. It inhibits platelet secretory activity and aggregation, maintains vasorelaxation, blocks monocyte-vascular wall interactions and is vasoprotective [14]. Because of its instability* in vivo*, several PGI_2_ analogs have been developed and demonstrated to reduce ischemic brain damage [15–17].

Our previous results have shown that overexpression of COX-1 and PGIS was able to generate large quantity of PGI_2_ in human endothelial cells [18]. Using adenovirus-mediated transfer of COX1 or COX1/PGIS, Lin et al., [19] and our coworkers [20] have demonstrated that enhanced PGI_2_ synthesis in neuronal cultures or in ischemic brain was neuroprotective and had prominent influence on microglia. This work attempts to selectively augment PGI_2_ synthesis in mixed glial culture and in hemiparkinsonian rats by direct adenoviral gene transfer of PGIS or bicistronic COX-1/PGIS and examines whether it confers protection or induces cell damage in Parkinson's disease (PD). Hemiparkinsonian rats were induced by injection of 6-OHDA to middle forebrain bundle (MFB), the ascending pathway of nigrostriatal system. Our results demonstrated that enhanced prostacyclin synthesis inhibited glial activation and was beneficial in hemiparkinsonian rats.

## 2. Materials and Methods

### 2.1. Materials

Lipopolysaccharide (LPS; *E. coli *0111:B4), OHDA, dopamine, apomorphine and methylthiazol tetrazolium (MTT) were obtained from Sigma-Aldrich (St. Louis, MO). CAY10449 was purchased from Cayman Chemical (Ann Arbor, MI). Cultured media and antibiotics were purchased from Invitrogen (Carlsbad, CA, USA). [6-^3^H] thymidine, [1-^14^C] arachidonic acid (AA) and radioactive prostanoids were purchased from Amersham Biosciences (Buckinghamshire, UK). Acetonitrile and other organic solvents were obtained from Merck (Darmstadt, Germany). Unless stated otherwise, all other chemicals were purchased from Sigma-Aldrich Co.

### 2.2. Recombinant Adenovirus (Ad)

Replication-defective first generation E1-deleted adenoviral vectors were used. Adenovirus encoding GFP (expressing the green fluorescence protein of jelly fish), PGI_2_ synthase (PGIS), or bicistronic COX-1/PGIS used phosphoglycerate kinase (PGK) as a driving promoter. The preparation, *ex vivo* expansion, and purification of these Ads followed methods described previously [18, 20, 21]. The viral titers of the purified Ads were determined by a plaque-forming assay and were in the range of ~10^10^ pfu/mL.

### 2.3. Cell Cultures and *In Vitro* Transduction

Mixed neuronal/glial cell cultures were prepared from the mesencephalic region of embryonic Sprague-Dawley (SD) rat fetus at gestation of 14–16 days as described in Tsai et al. [22, 23]. Briefly, cells were dissociated with mixtures of papain/protease/deoxyribonuclease I (0.1% : 0.1% : 0.03%) and plated onto poly-D-lysine coated dishes at a density of 1~2 × 10^5^ cells/cm^2^ in DMEM supplemented with 10% FBS. Second day after cell seeding, cultures were infected with AdGFP, AdCOX-1 or AdCOX-1/PGIS. Neuronal cells in the mixed neuron/glial cultures were identified by immunostaining against anti-*β*III tubulin (dilution 1/300; Covance, CA, USA). Mixed glial cells were prepared from neonatal rat brains as described previously [24, 25]. Briefly, triturated cortexes, free of vessels and meninges, were passed through nylon cloths and plated in 75 cm^2^ flasks in DMEM supplemented with 10% FBS. The cells were incubated at 37°C in a water-saturated atmosphere of 5% CO_2_/95% air. When cell reached confluence, cells in the flasks were subcultured and replated into multiwell plates. Cultures showed greater than 90% positive staining for glial fibrillary acidic protein (rabbit or mouse anti-GFAP, Chemi-Con, USA), an astroglial marker. Subconfluent cultures were used for measure of proliferative activity (tritiated thymidine incorporation or MTT reduction; see below), while confluent cultures were used for assay of metabolic activity in response to ^14^C-arachidonic acid (AA). For *in vitro *transduction, cells were fed with growth medium. Ad-GFP, Ad-PGIS, or Ad-COX-1/PGIS was added to cultured cells with a multiplicity of infection (MOI, pfu/cell) of 20. Recombinant Ad-GFP was used as a vector control and for optimizing the infection conditions. Three days after Ads transduction, cells were processed for eicosanoid measurement or treated with LPS (600 ng/mL) for 2 days. The culture medium was then assayed for nitric oxide (NO), and the cells were processed for western blot analysis. CAY10449 at 500 nM was added to mixed glial cells at 2 hr after AdPGIS transduction. Cultures were incubated for 5 days with medium and drug refilled once. Cultures were then processed for MTT reduction.

### 2.4. Extraction and Analysis of Arachidonic Acid (AA) Metabolites in Mixed Glial Culture and in Mixed Neuron/Glial Cultures

Confluent glial cultures or neuron/glial cultures after AdGFP, AdPGIS AdCOX-1, or AdCOX-1/PGIS transduction were measured for AA metabolic activity. Briefly, cultured cells were incubated in DMEM (serum-free) containing 10 *μ*M [1-^14^C] AA at 37°C for 10 min. The cells were saved for western blot analysis, while the released fractions, containing radioactive eicosanoids, were extracted by a Sep-Pak C_18_ cartridge (Waters Associates, Milford, MA) as described [20, 21]. The resulted extracts of ^14^C-labeled AA metabolites (eicosanoids) were analyzed by reverse phase high performance liquid chromatography (HPLC; Waters model 2690) equipped with an online radioisotope detector (Packard 150-TP) as previously described [18]. Briefly, the stationary phase was Inertsil 7 ODS-3 (4.6 × 150 mm; Vercopak, Taiwan). The mobile phase consisted of programmed gradient elution between solvent A (acetonitrile) and solvent B (0.1% acetic acid, pH 3.7) at a flow rate of 1 mL/min as follows: 34% B for 10 min, 34–40% B within 4 min, 40–50% B within 1 min, 50% B for 5 min, 50–75% B within 10 min, 75–100% B within 10 min, and 100% B for 10 min. The eicosanoids were identified by their retention times with the authentic radioisotope standards.

### 2.5. [^3^H]-Thymidine Incorporation Assay

The proliferative activity of mixed glial cultures after Ad transduction was investigated in subconfluent cultures by pulse of cultures with 0.5 *μ*Ci/mL [^3^H]-thymidine for 10 hrs according to our previous methods [26, 27]. After [^3^H]-thymidine pulse, cultured media were carefully removed and cells were washed twice with PBS. Aliquot of ice-cold 10% trichloroacetic acid (250 *μ*L/well) was added to cells. The radioactivities in the cell lysate were measured in a scintillation counter. 

### 2.6. 6-OHDA Lesion

Adult SD rats weighing 250–300 g were used. The animals were anesthetized by isoflurane (1-chloro-2,3,4-trifluoroethyl ether, Aerrane) with oxygen during surgery. Operations were carried out using an operating microscope under aseptic conditions. All procedures involving animals were approved by the Animals Committee of Taipei Veterans General Hospital. Surgical procedures, postoperative care, and monitoring have been described previously [28–30]. Unilateral 6-OHDA lesion of nigrostriatal pathway was performed with the rats under isoflurane anesthesia by stereotaxic injection of 6-OHDA HBr [20 *μ*g/rat; dissolved in ascorbate (0.02% in PBS as vehicle)]. Five microliters of 6-OHDA (2 *μ*g/*μ*L) were injected into two sites (5 *μ*L/site) in the ascending nigrostriatal pathway near the MFB of adult SD rats as described [31]. The coordinates of two MFB injections were AP-4.2 mm (posterior to bregma), ML-1.1 mm (lateral to the midline), DV-7.8 mm (7.8 mm below the dura), AP-4.4 mm (posterior to bregma), ML-0.9 mm, and DV-7.8 mm. The needle was allowed to remain in the brain for 5 min before being retracted at the end of the 6-OHDA infusion.

### 2.7. Intranigral Injection of Ad Vectors

After the rat was placed in the stereotaxic frame (Kopf Instruments, Tujunga, CA) infused with saline or 6-OHDA, injection of Ad vector to brain regions was conducted. 1 *μ*L Ad vector suspended in PBS was injected into the vicinity of the SN at [coordinate AP −5.3 mm, ML −2.1 mm, and DV −7.2 mm from bregma] or into striatum at [AP+0.5 mm; ML+2.0 mm and DV−5.0 mm from bregma]. Ad injection was through a 5 *μ*L Hamilton syringe fitted with a 30-gauge beveled hypodermic needle for 5 min at a rate of 0.2 *μ*L/min. After the cessation of the injection, the needle was left in place for 5 min before being slowly withdrawn from the brain. Ad vector injection was conducted within 30 min after infusion of 6-OHDA to MFB.One microliter of storage buffer, Ad-GFP, or AdCOX-1/PGIS containing approximately 2 × 10^4^ plaque-forming units (pfu) was injected. AdGFP was used as a mock control and for examining infective tropism. 

### 2.8. Apomorphine-Induced Circling Behavior

 One to four weeks after the infusion of 6-OHDA and Ads, a behavioral test was conducted to identify the efficacy of treatment. Rats from all groups were administered with the DA agonist apomorphine (0.5 mg/kg, s.c.) and immediately separated into individual acrylic box cages. Ten minutes later, the number of contralateral rotations to the lesioned side was recorded in each rat every 5 min for a total time of 60 min. Each rotation was defined as a complete 360° turn. Results were expressed as the total number of turns that rats completed in 60 min. In the present study, rats with 6-OHDA lesion only would have a net contralateral rotational asymmetry of >500 turns/hr.

### 2.9. Biochemical Assays

The production of nitric oxide (NO), as nitrite accumulation, was assayed in the medium using colorimetric reaction with Griess reagents (1% sulfanilamide/0.1% naphthyl ethylene diamine dihydrochloride/2% phosphoric acid) as described [23]. The degree of MTT reduction was used to measure cell viability or proliferative activity. Following treatment, MTT was added to cultured cells at final concentration of 0.1 mg/mL and reacted with the surviving cells for 4 hr at 37°C. The resulted blue formazan product was solubilized and measured at an absorbance of 570 nm [26]. 

### 2.10. Immunohistochemistry

Rats were perfused intracardially with 4% paraformaldehyde in PBS under deep anesthesia with sodium pentobarbital at the end of the experiments. The brains were removed, postfixed in 4% paraformaldehyde overnight, and then cryoprotected in PBS containing 30% (w/v) sucrose for 3 days. The tissues were excised and embedded in Tissue Tek OCT (Sakura Fine Technical, Tokyo, Japan) and then cross-sectioned at 20 *μ*m thickness with a cryostat. Tissue sections were collected onto glass slides and dried at 37°C. The tissue sections were incubated with primary antibodies, followed by respective 2nd antibodies for histological evaluation as described [30, 32]. To examine the infective tropism of AdGFP, rats were processed for histological analysis at three days after nigral AdGFP infection. Double immunostaining of GFP with cell markers was conducted in coronal brain sections. Antibodies for cell markers included anti-tyrosine hydroxylase (TH) (mouse, dilution 1/150, Chemicon, Temecula, CA) for dopamine neurons, anti-GFAP (rabbit, dilution 1/1000; Chemicon, Temecula, CA) for astrocytes, Biotinylated *Griffonia simplicifolia* lectin I isolectin B4 (biotinylated GSLI-IB4, dilution 1/100; vector b1205), or anti-ED-1 (mouse, dilution 1/250; Serotec, Oxford, UK) for microglia. 

### 2.11. Western Blot Analysis

Brain tissues or cultured cells were solubilized in lysis buffer containing 7 M urea, 2 M thiourea, 4% CHAPS, 40 mM Tris buffer, pH7.5, protease inhibitors (Roche, Mannheim, Germany), 1 mM PMSF, 1 mM Na_3_VO_4_, and 1 mM DTT. Protein concentration of the resultant lysate was determined using Bio-Rad protein assay (Bio-Rad, Hercules, CA). Equal amounts of proteins were loaded and separated using 8%–12% gels (SDS-PAGE) as described [33]. After electrophoresis, proteins in the gels were transferred to PVDF membranes (Millipore Corp., USA) and incubated overnight at 4°C with antibodies against PGIS (rabbit, dilution 1/3000), COX-2 (rabbit, dilution 1/5000, Cayman), inducible nitric oxide synthetase (iNOS, mouse, dilution 1/5000, BD Bioscience, USA), or *β*-actin (goat, dilution 1/5000, Santa Cruz Biotech, USA) followed by a horseradish peroxidase-conjugated secondary antibody (dilution 1/2000, Jackson Lab) for 1 hr at room temperature. Immunoreactivity was visualized by enhanced chemiluminescent detection (Perkin Elmer Co., USA).

## 3. Results 

### 3.1. Analysis of Eicosanoids Produced by Cultured Glial Cells in Response to [1-^14^C] AA Treatment

**Figure 1:**
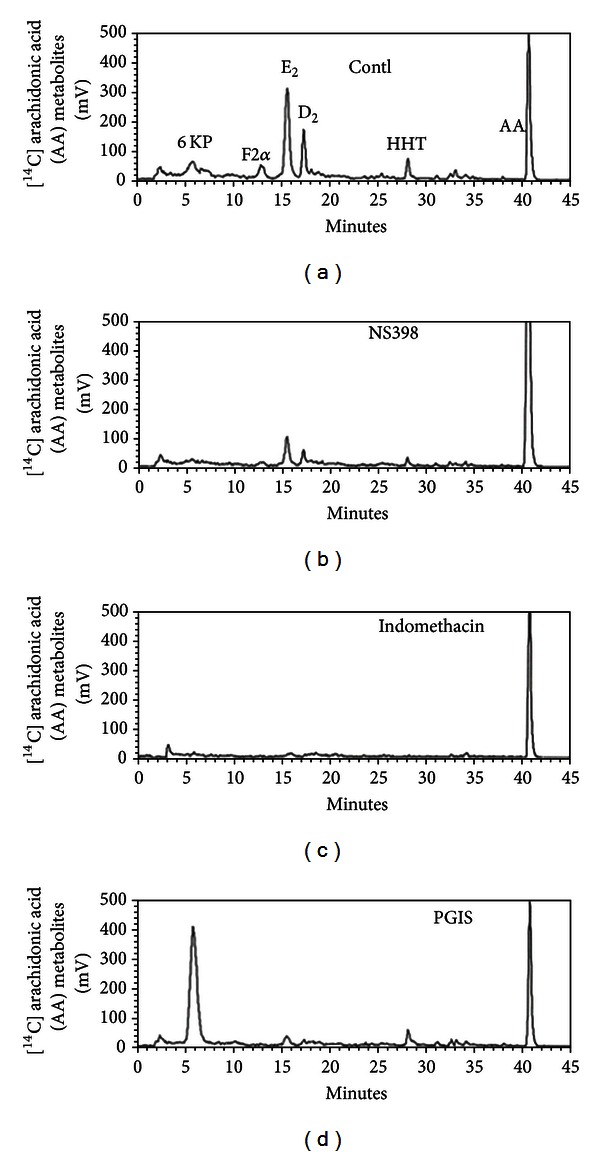
Analysis of ^14^C-labelled eicosanoids generated in control or Ad-PGIS-transduced mixed glial cultures in response to [1-^14^C] AA and COX inhibitors. Measurement of eicosanoid biosynthesis in cultures was conducted at 3 days after Ad-PGIS transduction. NS398 is a COX-2 specific inhibitor, and indomethacin is an inhibitor for both COX-1 and COX-2. Inhibitor was added to cultures 30 min before and during ^14^C-AA pulse. 6-KP denotes 6-keto-PGF_1*α*_, the product of PGI_2_ hydrolysis. Peaks of first 5-minute fractions are nonspecific. Each prostanoid peak was verified by coelution with an authentic radiolabelled prostanoid.

Purified Ads were successfully expanded and purified before conducting the* in vitro* and *in vivo* experiments. Highly purified adenoviruses encoding GFP, PGIS, and bicistronic COX-1/PGIS, ranging from 10^10^~10^11^ pfu/mL, were used in the present study. Previously, we have demonstrated high permissivity of mixed glial cultures to AdGFP infection [22]. Almost all cells expressed GFP at 2 days after 20 MOIs of Ad-GFP transduction (Similar results are shown in Figure 4(f)). In the present study, we directly examined the activities of eicosanoid biosynthetic activity in Ad-transduced mixed glial cells in response to ^14^C-AA pulse. Because mixed glial cultures are depleted of neuronal cells, studying AA metabolic activity in glia cultures would give some clue to the relative roles of neurons versus glial cells. We incubated cultures with [1-^14^C] AA for 10 min, extracted eicosanoids from the medium by a C_18_ cartridge, and analyzed the eicosanoids by HPLC. Two predominant peaks, prostaglandin (PG) E_2_ and PGD_2_, were detected in nontreated cultures (Figure 1(a)). Very little or none of PGI_2_ (prostacyclin), shown as its hydrolysis product 6-keto-PGF_1*α*_ (6KP), was found. The transduction of AdGFP did not alter the metabolic profile (data not shown). The 6-keto-PGF_1*α*_ peak was mostly reduced (>80%) when cells were pretreated with NS398, a selective COX-2 inhibitor (Figure 1(b)). Furthermore, no 6-keto-PGF_1*α*_ peak was detected when cells were pretreated with indomethacin, an inhibitor for both COX-1 and COX-2. This indicates COX-2 as the major enzyme of eicosanoid synthesis in mixed glial cultures in response to ^14^C-AA. Interestingly, AA metabolites were shunted through prostacyclin synthesis on AdPGIS transduction. Very little of PGE_2_ and PGD_2_ remained in AdPGIS-transduced cultures. This indicates that the overexpressing enzyme was functionally active in producing prostacyclin from AA. By contrast, AdPGIS-infected neuron/glial cultures did not augment 6-keto-PGF_1*α*_ synthesis (see Supplementary Figure 1 available online at http://dx.doi.org/10.1155/2013/649809 and Tsai et al., [20]). AdCOX-1-infected neuron/glial cultures produced predominant PGE_2_ and PGD_2_ peaks. Only bicistronic AdCOX-1/PGIS-infected neuron/glial cultures prominently enhanced 6-keto-PGF_1*α*_ synthesis. 

### 3.2. Enhanced Prostacyclin Synthesis Reduced Cell Proliferation and LPS Stimulation in Mixed Glial Cells

**Figure 2:**
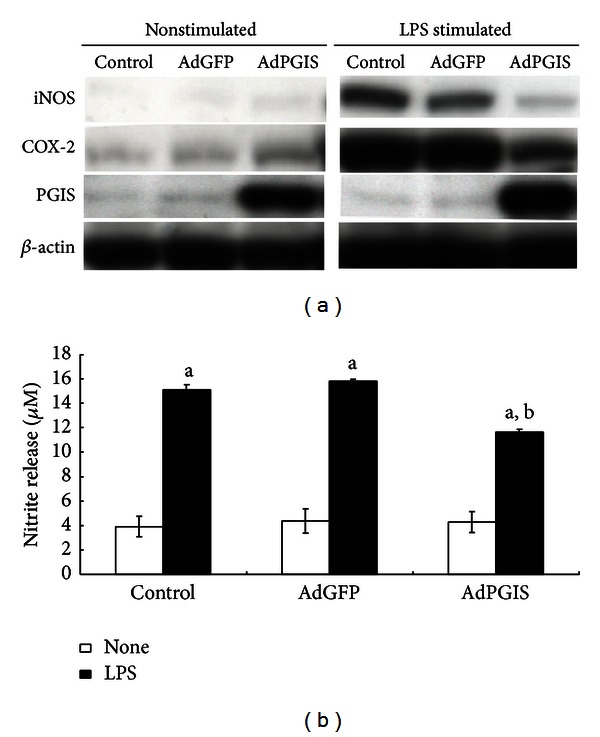
NO synthesis and expressions of iNOS, COX-2, and PGIS in AdGFP- and AdPGIS-transduced mixed glia cultures before and after LPS challenge. (a) Western blot analysis of protein levels of PGIS, COX-2, and iNOS in cultures receiving AdGFP or AdPGIS infection. After 3 days of Ad infection, cultures were treated with LPS (600 ng/mL) for 2 days and harvested. Equal amounts of protein were analyzed by western blot using anti-PGIS, anti-COX-2, and anti-iNOS antibodies. Protein bands were visualized using horseradish peroxidase-conjugated secondary antibodies and electrochemiluminescence (ECL). (b) Inhibition of LPS-stimulated NO production, as nitrite release, in the medium by AdGFP and AdPGIS transduction in cultures. Control on *x*-axis, no viral infection of naive cultures; open bar in chart indicates nonstimulated cultures. Closed bar indicates LPS-stimulated cultures. Data were expressed as means ± SEM from four independent experiments done in triplicate. ^a^ *P* < 0.01 indicates significant differences between nonstimulated and LPS-stimulated cultures within each Ad-transduced cells; ^b^ *P* < 0.05 AdPGIS + LPS compared with AdGFP + LPS.

**Figure 3:**
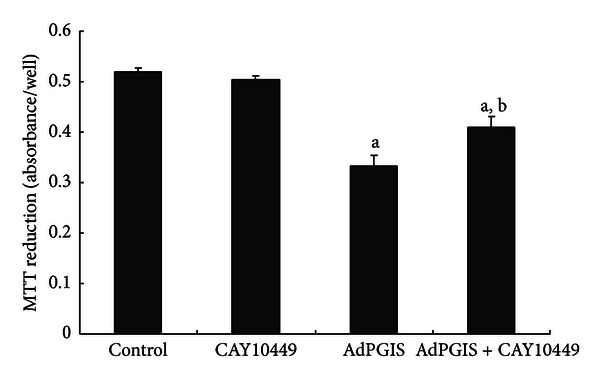
CAY10449 and AdPGIS transduction on MTT reduction in mixed glial cultures. CAY10449 is a high affinity ligand and functional antagonist for the human IP (prostacyclin) receptor. CAY10449 (500 nM) was added to cultured cells at 2 hr after AdPGIS transduction. Data were means ± SEM from 4 independent cultures done in duplicate. ^a^ *P* < 0.01 AdPGIS versus Control; ^b^ *P* < 0.05 AdPGIS + CAY10449 versus AdPGIS.

To examine the effects of enhanced prostacyclin synthesis on cell proliferation, subconfluent glial cells were transduced with Ad-PGIS for 2 days. Tritiated thymidine was added to cultures 10 hr before cell harvest.  Table 1 shows that overexpression of PGIS in cultured glial cells concurrently enhanced prostacyclin production (indicated by level of 6-keto-PGF_1*α*_, the product of PGI_2_ hydrolysis) and inhibited astroglial proliferation (indicated by thymidine incorporation). To examine whether the effect of overexpressing PGIS on cell proliferation was mediated via IP (prostacyclin) receptor, CAY10449 was added to mixed glial cells at 2 hr after AdPGIS transduction. As shown in Figure 2, AdPGIS transduction reduced degree of MTT reduction in mixed glial cultures. The high-affinity IP antagonist, CAY10449, at 500 nM partially but significantly abrogated prostacyclin effect on cell proliferation. LPS is a powerful immune challenge. LPS treatment has been shown to induce release of cytokines, NO, and proinflammatory factors from macrophages [34, 35]. Effect of overexpressing PGIS on LPS stimulation was examined in mixed glial cultures. Confluent glial cultures were transduced with AdGFP or AdPGIS. Three days later, cells were further treated with LPS at a dose of 600 ng/mL for 2 days. In our previous paper [20], we used 100 ng/mL LPS to stimulate neuron/glial cultures. However, LPS failed to affect mixed glial cells at 100 ng/mL and required higher concentration of LPS for effective cell stimulation. As shown in Figure 3, Ad-PGIS transduction enhanced the expression of PGIS whereas iNOS and COX-2 levels were barely detectable. By contrast, LPS treatment induced increase of iNOS and COX-2 expression in control and AdGFP-infected cells. AdPGIS transduction effectively reduced LPS-induced COX-2 and iNOS levels. Concurrently, LPS-stimulated nitrite releases were significantly inhibited in AdPGIS-transduced cells (Figure 3(b)) compared to control and Ad-PGK-infected cells. This indicates that enhanced prostacyclin synthesis in PGIS-overexpressing cells significantly inhibited iNOS expression and NO production.

### 3.3. Infective Tropism of AdGFP Transduction in Mesencephalic Neuron/Glial Cultures and in Rat Substantia Nigra (SN)

**Figure 4:**
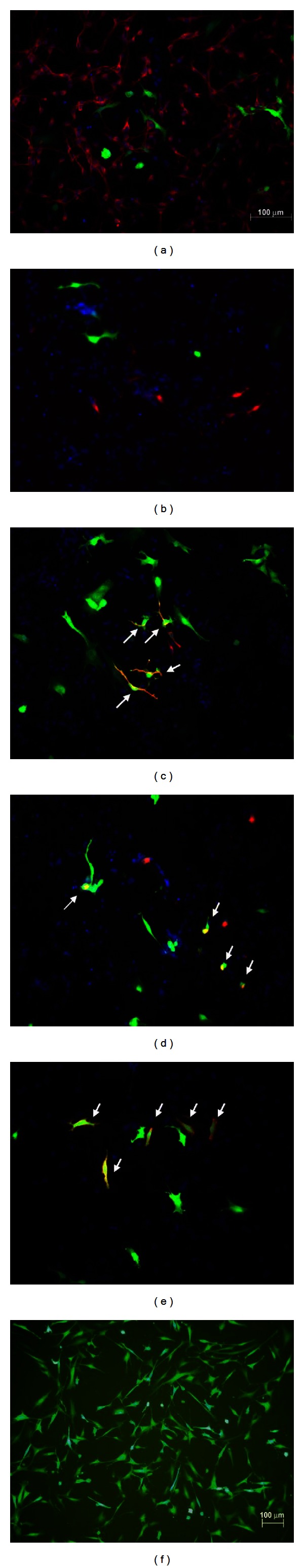
Infective tropism of adenovirus encoding GFP (AdGFP) in mesencephalic neuron/glial cultures and GFP expression in AdGFP-infected mixed glial cells. (a)~(e) are representative micrographs of double labeling staining of cultures maintained in serum-free condition; Green color: GFP-immunoreactivity (IR). Red color in (a) *β*III tubulin-IR for neurons, (b) tyrosine hydroxylase (TH)-IR for dopamine neurons, (c) GFAP-IR for astroglia, (d) ED1-IR for microglia, (e) NG2-IR, Magnification ×200, (f) mixed glial cells, magnification ×100. Arrows or arrow head in the figure indicates double-staining IR. AdGFP (10^6^ pfu/well each) was added to cultured cells in serum or serum-free medium. The infective tropism of AdGFP in serum-free condition was 19.6 ± 2.9% GFAP(+) cells, 24.4 ± 6.1% ED1(+) cells, and 43.9 ± 4% NG2(+) cells. GFP(+) cells in neuron/glial cultures in serum-free and serum-containing conditions during infection were 31.83 ± 3.12 and 19.89 ± 2.19 cells/mm^2^, respectively.

**Figure 5:**
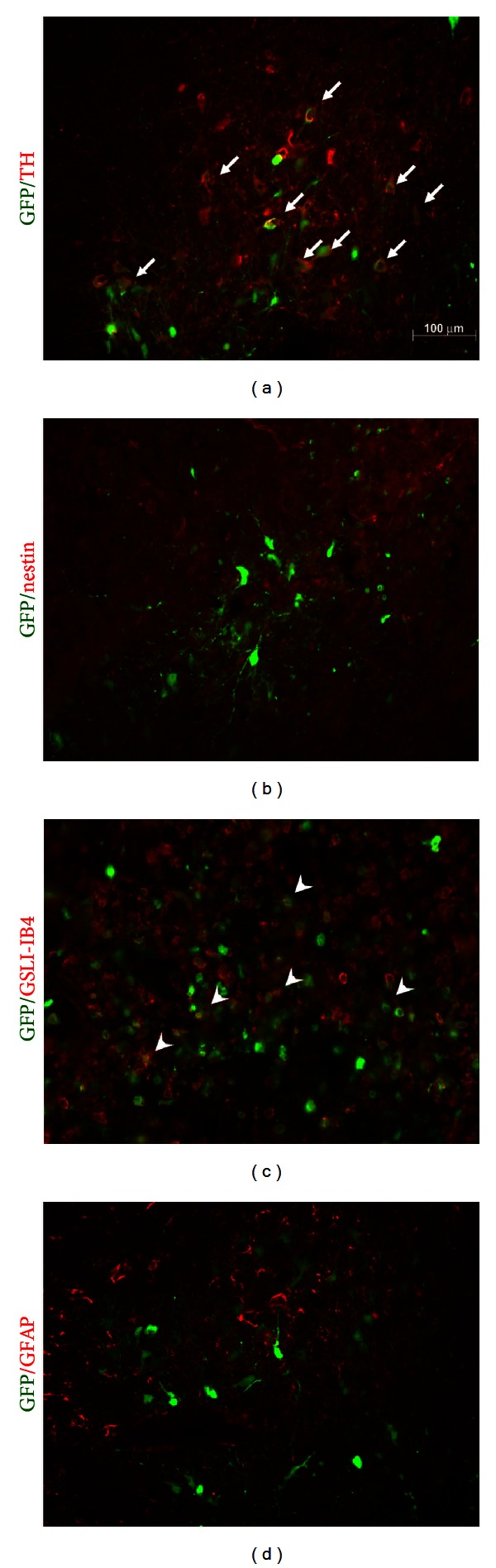
Infective tropism of adenovirus encoding GFP (AdGFP), which was injected into the substantia nigra (SN) 3 days ago. Representative micrographs of double labeling staining of coronal sections in the injection site. (a)~(d) Photos (magnification ×200) of each double staining; green color: GFP immunoreactivity (IR); red color: TH-IR (panel a), nestin-IR (panel b), GSLI-IB4-IR (panel c) and GFAP (panels d). Arrows or arrow heads in the figures indicate double-IR. AdGFP could transduce dopaminergic (TH-IR) neurons and GSLI-IB4-IR microglia in the SN.

First, we examined the infective tropism of AdGFP in mesencephalic neuron/glial cultures which were enriched with DA neurons. AdGFP (~10^6^ pfu/well each) was added to cultured cells in serum or serum-free medium. As shown in Figure 4, AdGFP predominantly transduced nonneuronal cells. In serum-free condition, the AdGFP infective cells were 19.6 ± 2.9% astroglial (GFAP-positive) cells, 24.4 ± 6.1% microglia (ED1-positive) cells, and 43.9 ± 4% NG2-positive cells. No *β*III tubulin-positive neuron or TH-positive DA neurons showed GFP immunoreactivity. Furthermore, the AdGFP infective efficiency in serum-containing medium (10% FCS) was reduced compared to that in serum-free condition. Using same titer of AdGFP to neuron/glial culture, GFP-positive cells in cultures maintained in serum-free and serum-containing conditions were 31.83 ± 3.12 and 19.89 ± 2.19 cells/mm^2^, respectively. We further performed double-labelled staining for AdGFP-infected coronal sections containing SN. After injection of AdGFP into the normal SN, the expression of GFP was observed along the site of injection.  Figure 5 shows the representative micrographs of double-labelled immunostaining results. Some GFP-positive cells in the SN were also positively stained for TH, which denote dopaminergic neurons (Figure 5(a), arrow). Some GSLI-IB4-positive microglia (in Figure 5(b), arrow head) seemed to be immunoreactive to GFP. By contrast, almost no double-labelled staining cells were found in GFP with GFAP or nestin immunoreactivity (Figures 5(c) and 5(d)). These results showed that transgene expression could be achieved in neurons or microglia after injection of AdGFP into the SN *in vivo*. The results of AdGFP tropism in cultures and in SN were not consistent. We also infected AdGFP to the striatum and examined the infective tropism (shown in Supplementary Figure 2). Consistent with the results observed in AdGFP-infected SN* in vivo*, TH-positive, GFAP-positive, ED1-positive and nestin-positive cells were found to be double labelled with GFP in rat striatum. 

### 3.4. Effect of Nigral Infusion of Ad-COX-1/PGIS in Hemiparkinsonian Rats

**Figure 6:**
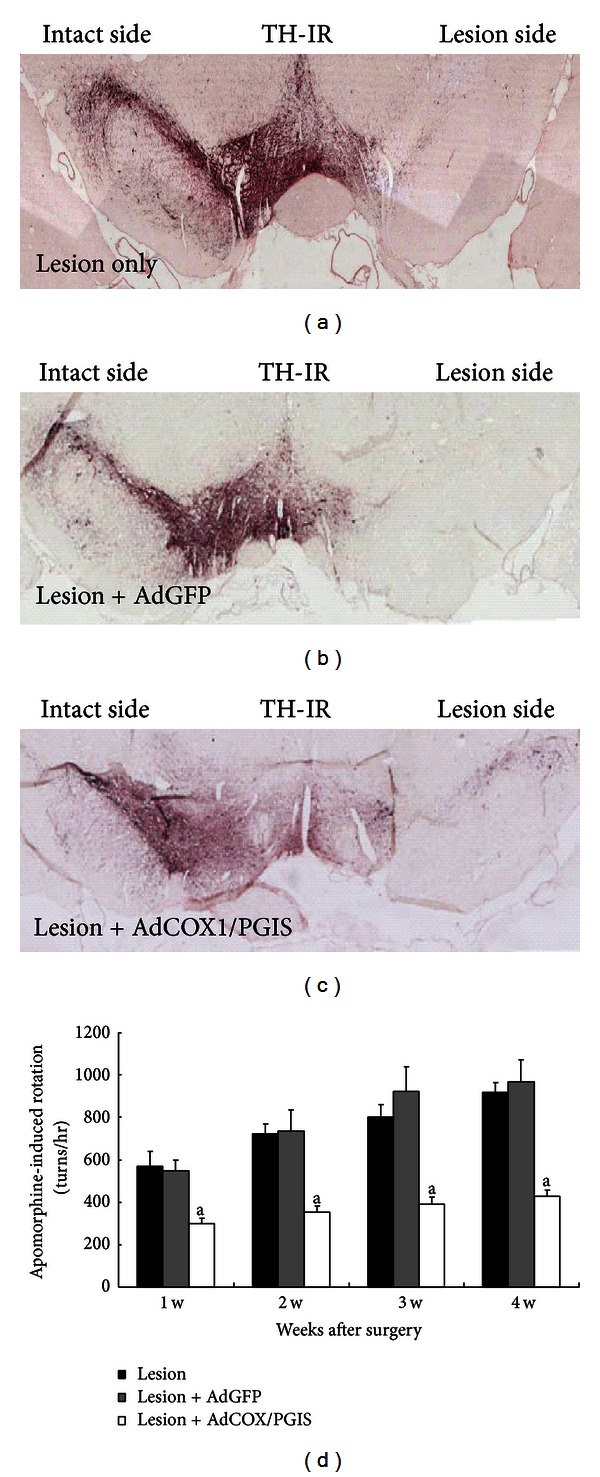
Nigral Ad-COX-1/PGIS infusion ameliorated 6-OHDA-induced dopamine neuronal loss and behavioral deficit. (a)~(c) Representative micrographs of TH immunoreactivity in rat substantia nigra (SNpc) of each group. 20 *μ*m thick coronal brain sections, In (a) 6-OHDA-lesioned brain section, (b) 6-OHDA-lesioned + AdGFP-transduced brain section and (c) 6-OHDA-lesioned + AdCOX1/PGIS-transduced brain section. (d) Apomorphine-induced rotational behaviors in 6-OHDA-treated rats. TH-positive neurons in (c) were rescued by AdV-COX1/PGIS adenoviral gene transfer. 6-OHDA treatment severely depletes TH-positive neurons in right SN compared with untreated left site. Adenoviral vectors were injected into SN within 30 min after 6-OHDA treatment. Treatment rescued TH-positive neurons ipsilateral to gene delivery. In all cases, Ad treatment had no effect on the contralateral SNpc. Data are means ± SEM from 8, 5, and 13 rats for Lesion, Lesion + AdGFP, and Lesion + AdCOX-1/PGIS groups, respectively. ^a^ *P* < 0.01 compared to lesion only at the same time.

Our previous study showed that bicistronic AdCOX1/PGIS infection to neuron/glial cultures produced prominent prostacyclin synthesis, whereas AdPGIS infection did not [20]. We thus directly infused AdCOX-1/PGIS to rat SN for ensured prostacyclin production. 6-OHDA at a dose of 20 *μ*g/rat was infused to the right MFB in rats. Within 30 minutes after 6-OHDA infusion, AdGFP or AdCOX1/PGIS (2 × 10^4^ pfu) was subsequently infused to right SN. Behavioral test was examined weekly after surgery by apomorphine-induced turning. Rats were sacrificed 4 weeks after treatment. Regions of substantia nigra and striatum were microdissected for dopamine level measurement. Figures 6(a) and 6(b) demonstrate 6-OHDA treatment severely depleted TH-positive neurons in right SN compared with untreated left site. Some TH-positive neurons were rescued by AdCOX1/PGIS gene transfer (Figure 6(c)). Treatment rescued TH-positive neurons ipsilateral to gene delivery. In all cases, Ad treatment had no effect on the contralateral SN. Apomorphine-induced turning behaviors, as a marker of motor impairment, in 6-OHDA-lesioned rats were shown in Figure 6(d). 6-OHDA-treated rats displayed a considerable number of rotations. And this effect was significantly reduced in rats transduced with AdCOX1/PGIS (*P* < 0.01). The group treated with AdGFP transduction produced no significant changes in rotation behavior when compared with 6-OHDA lesion only. Taken together, nigral Ad-COX-1/PGIS infection protected against 6-OHDA-induced dopaminergic damage and behavioral deficits. 

## 4. Discussion

Adenovirus-mediated gene transfer is a promising tool for the treatment of neurodegenerative diseases. Recombinant adenoviral vectors target gene expression to the nervous system and offer prolonged expression of foreign proteins [23, 33, 36]. Here, we present evidence that glial cultures, mesencephalic neuron/glial cultures, or nigral dopaminergic neurons (SNpc) could effectively be infected by recombinant adenoviruses and thereby expressing transgenes. Our data with glial cultures demonstrated that unstimulated cells actively metabolized exogenous AA into PGE_2_ and PGD_2_. These PGs were largely NS398 sensitive and therefore considered as COX-2-derived products. This was in contrast to low AA metabolic activity in neuronal cells, which released barely detected eicosanoids [20]. Interestingly, gene transfer of Ad-PGIS to glial cultures effectively shunted the AA catabolism through synthesis of 6-keto-PGF_1*α*_, a hydrolyzed product of prostacyclin. This indicates that the overexpressing enzyme was functionally active in cooperation with endogenous COX to produce prostacyclin. Accordingly, enhanced prostacyclin production effectively inhibited glial proliferation, in part, through prostacyclin IP receptor. Furthermore, enhanced prostacyclin synthesis significantly inhibited LPS stimulation through inhibition of COX-2 and iNOS expression and NO production. Cell viabilities, measured as LDH release in medium, were not affected by Ad infection or LPS treatment (data not shown). 

It is well known that LPS, a major component of the outer membrane of Gram-negative bacteria, induces inflammatory response through cytokine production [37]. We found that mixed glial cells were less sensitive to LPS stimulation in comparison to mixed neuron-glial cultures. LPS is a powerful tool for the activation of microglia. Although LPS has no known direct toxic effect on neurons, it activates microglia to release neurotoxic factors [38, 39]. Neurons, through mechanisms of cell-cell interaction, also modulate the reactivity of microglia [40, 41]. In the present study, AA metabolic activities in neuron/glial culture and in glial cultures were quite different, highlighting the involvement of cell-cell interaction. This work aims to examine the effect of PGI_2_ in nervous system. Our previous results have shown that overexpression of COX-1 and PGIS is able to generate large quantity of PGI_2_ [18]. AdCOX-1 infected cells could overexpress COX-1 with consistent high activity (mainly PGE_2_), as shown in Supplementary Figure 1 in which ^14^C-labelled eicosanoids were generated by Ad COX-1-infected neuron/glial cultures in response to [1-^14^C] AA treatment. Therefore, we used the Ad-COX-1/PGIS to investigate the effect of enhanced PGI_2_ synthesis in substantia nigra* in vivo*. Both COX-1 and COX-2 catalyze conversion of AA to PGG_2_ and further convert to PGH_2_. It is possible that overexpression of COX-2/PGIS will generate large quantity of PGI_2_ as well.

Our data with adenoviral gene transfer in rat SN indicate that recombinant adenovirus could infect both dopaminergic neurons and microglia efficiently. Among GFP-expressing cells in SN, 58.7 ± 4.8% were TH-positive dopamine neurons and 24.3 ± 6.4% were microglia. The transgene expression was not found in GFAP- or nestin-positive cells, in contrast to that seen in mesencephalic neuron/glial cultures. Figure 4 shows that AdGFP preferentially infected NG2(+) cells, ED1(+) and GFAP(+) cells, while no beta III tubulin (+) neurons or TH(+) dopamine neurons were infected in mixed neuron/glial cultures. High permissivity to AdGFP transduction in mixed glial cells was also shown. Because mixed glial or neuron/glial cultures were maintained as monolayer cultures, nonneuronal cells in these cultures extended and flattened, allowing high probability of getting AdGFP infection. While SN is enriched with DAergic cell body and particularly rich in microglia [2, 3], targeting AdGFP injection to SN *in vivo* was possible to be taken up by microglia and by large area of DA dendrites *in situ*. Thus, rare GFAP/GFP double-labelled cells could be found. Our previous study showed that bicistronic AdCOX1/PGIS infection to neuronal cultures produced prominent prostacyclin synthesis [20]. This was also true in endothelial cells that the overexpressed PGIS and COX-1 were colocalized, leading to monophasic overexpression of prostacyclin [21]. We thus directly infused AdCOX-1/PGIS to rat SN for ensured prostacyclin production.  Figure 6 showed that overexpression of COX1/PGIS reduced dopaminergic neuronal death induced by 6-OHDA. We provide evidence that a decrease of the number of TH-positive cells at 4 weeks after 6-OHDA lesion corresponds well with rotational behavior. 6-OHDA-treated rats displayed a considerable number of rotations. This effect was significantly attenuated in rats transduced with AdCOX1/PGIS (*P* < 0.01). The 6-OHDA lesion group with AdGFP transduction produced no significant changes in rotation behavior when compared with 6-OHDA lesion only. Taken together, nigral Ad-COX-1/PGIS infection ameliorated 6-OHDA-induced dopaminergic damage and behavioral deficits.

We used first-generation E1-deleted Ad vectors in the present study. Possible neurotoxic effects induced by Ad expression should be considered. Although we did not evaluate immune response in this study, there were no significant side effects in rats with Ad injection. Furthermore, AdPGIS, AdCOX-1/PGIS or AdGFP used in this study had been constructed with a PGK promoter that could drive prolonged (days to weeks) transgene expression. The mechanisms by which AdPGIS-transduction reduced glial activation or AdCOX1/PGIS-transduction reduced Parkinsonian dysfunction have not yet been clarified. In the brain, at least two distinct prostacyclin receptors, designated as IP1 and IP2, have been shown [42]. The IP2 receptor was found only in the CNS and thus named as central-type prostacyclin receptor [42]. The IP1 receptor is mainly coupled to G-protein-coupled receptor. Stimulation of the receptor results in cAMP production [43]. It remains to be determined which receptor subtype (IP1 or IP2) is involved in this effect. Further studies are needed to elucidate the role played by downstream molecules in the inhibition of cell proliferation and LPS stimulation and in protection to hemiparkinsonism. 

In conclusion, this study shows that enhanced prostacyclin synthesis by adenovirus-mediated gene transfer of AdPGIS or AdCOX1/PGIS reduced glial activation and ameliorated motor dysfunction in hemiparkinsonian rats. We suggest that prostacyclin may have a neuroprotective role in modulating the inflammatory response in degenerating nigra-striatal pathway.

## Supplementary Material

Supplementary figures demonstrate AA catabolic activity in Ads-infected neuron/glial cultures and Ad infective tropism in the striatum *in vivo*.
Supplemental Figure 1: Changes of AA catabolic activity in neuron/glial cultures overexpressing PGIS, COX-1 or COX-1/PGIS.
Supplemental Figure 2: Adenoviral infective tropism in rat striatum.

## Figures and Tables

**Table 1:** Effect of Ad-PGIS gene transfer on the proliferative activity in mixed glial cells.

	Thymidine incorporation	[^14^C] 6-keto-PGF_1*α*_
	(cpm/well)
Control	378 ± 19	100 ± 4
Ad-PGK	372 ± 24	103 ± 2
Ad-PGIS	277 ± 16*	750 ± 5**

Subconfluent rat glial cells in 24-well plates or 6 cm dishes were infected with Ad (5 × 10^5^ pfu/w) for 2 days. [^3^H]-thymidine (0.5 *μ*Ci) was added to culture (in 24-well plate) 10 hrs before cell harvest. Trichloroacetic-acid-(TCA-) insoluble fraction (radioactivity) was collected and counted. For glial cells grown in 6 cm dishes, cells were incubated with 10 *μ*M [1-^14^C] AA for 10 min. [1-^14^C] AA metabolites in the medium were then processed for HPLC analysis. Data are expressed as means ± SEM from 4 independent repeats. *P < 0.05; **P < 0.01 by one way ANOVA.
