# Supplementary material for: Enhanced Prostacyclin Synthesis by Adenoviral Gene Transfer Reduced Glial Activation and Ameliorated Dopaminergic Dysfunction in Hemiparkinsonian Rats
Source: Oxid Med Cell Longev. 2013 Apr 3;2013:649809. doi: 10.1155/2013/649809 (PMC3649752; doi:10.1155/2013/649809)
Supplement: Supplementary file 1 — Supplementary figures demonstrate AA catabolic activity in Ads-infected neuron/glial cultures and Ad infective tropism in the striatum in vivo. Supplemental Figure 1: Changes of AA catabolic activity in neuron/glial cultures overexpressing PGIS, COX-1 or COX-1/PGIS. Supplemental Figure 2: Adenoviral infective tropism in rat striatum. [file 649809.f1.doc]

**Supplement Figures:**


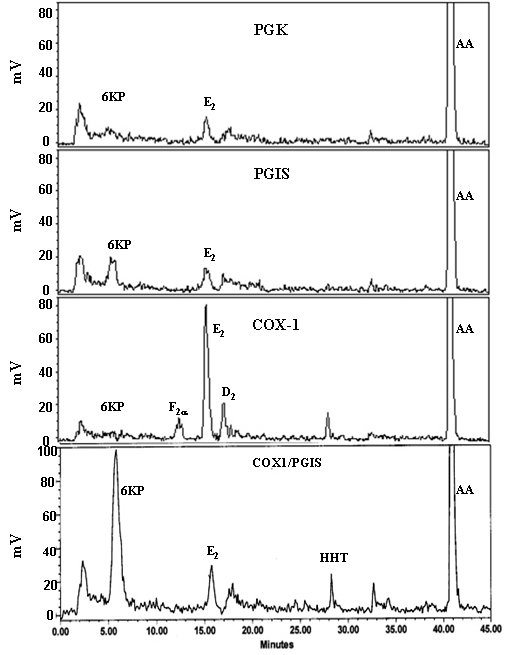


**FIGURE 1**: Analysis of ^14^C-labelled eicosanoids generated in Mock (PGK), AdPGIS-, AdCOX-1-or AdCOX-1/PGIS-transduced neuron/glial cultures in response to [1-^14^C] arachidonic acid (AA). Measurement of eicosanoid biosynthesis in cultures was conducted at 3 days after adenoviral transduction. Cultures were pulsed with ^14^C-AA for 10 min. The medium was then collected for extraction of radioactive eicosanoids. 6-KP denotes 6-keto-PGF_1α_, the product of PGI_2_ hydrolysis. Peaks of first 5-minute fractions are nonspecific. Each prostanoid peak was verified by coelution with an authentic radiolabelled prostanoid.

FIGURE 2: Infective tropism of adenovirus encoding GFP (AdGFP) in rat striatum. Representative micrographs of double labelling staining of coronal sections in the injection site (A)~(D) Photos (magnification x 200) of each double staining; Green color: GFP-immunoreactivity (IR); red color: TH-IR (panel A), nestin-IR (panel B), GSLI-IB4-IR (panel C) and GFAP (panels D). Arrows or arrow heads in the figures indicate double-labelling staining. AdGFP could transduce dopaminergic (TH-IR) neurons, GSLI-IB4-IR microglia, nestin-IR progenitor and GFAP-IR astroglia in the striatum *in vivo*.
